# Independent Activity of the Homologous Small Regulatory RNAs AbcR1 and AbcR2 in the Legume Symbiont *Sinorhizobium meliloti*

**DOI:** 10.1371/journal.pone.0068147

**Published:** 2013-07-15

**Authors:** Omar Torres-Quesada, Vicenta Millán, Rafael Nisa-Martínez, Florian Bardou, Martín Crespi, Nicolás Toro, José I. Jiménez-Zurdo

**Affiliations:** 1 Grupo de Ecología Genética de la Rizosfera, Estación Experimental del Zaidín, Consejo Superior de Investigaciones Científicas (CSIC), Granada, Spain; 2 Institut des Sciences du Végétal (ISV), CNRS, Gif-sur-Yvette, France; University of Padova, Medical School, Italy

## Abstract

The legume symbiont *Sinorhizobium meliloti* expresses a plethora of small noncoding RNAs (sRNAs) whose function is mostly unknown. Here, we have functionally characterized two tandemly encoded *S. meliloti* Rm1021 sRNAs that are similar in sequence and structure. Homologous sRNAs (designated AbcR1 and AbcR2) have been shown to regulate several ABC transporters in the related α-proteobacteria *Agrobacterium tumefaciens* and *Brucella abortus*. In Rm1021, AbcR1 and AbcR2 exhibit divergent unlinked regulation and are stabilized by the RNA chaperone Hfq. AbcR1 is transcribed in actively dividing bacteria, either in culture, rhizosphere or within the invasion zone of mature alfalfa nodules. Conversely, AbcR2 expression is induced upon entry into stationary phase and under abiotic stress. Only deletion of AbcR1 resulted into a discrete growth delay in rich medium, but both are dispensable for symbiosis. Periplasmic proteome profiling revealed down-regulation of the branched-chain amino acid binding protein LivK by AbcR1, but not by AbcR2. A double-plasmid reporter assay confirmed the predicted specific targeting of the 5′-untranslated region of the *livK* mRNA by AbcR1 *in vivo*. Our findings provide evidences of independent regulatory functions of these sRNAs, probably to fine-tune nutrient uptake in free-living and undifferentiated symbiotic rhizobia.

## Introduction

*Sinorhizobium meliloti* is a representative of the group of soil-dwelling α-proteobacteria, collectively known as rhizobia, which have the ability to engage in species-specific nitrogen-fixing endosymbioses with leguminous plants. The outcome of these mutualistic plant-microbe interactions is the formation of root nodule structures in the host. Within nodules invading bacteria undergo a morphological differentiation to bacteroids that are accommodated intracellularly to reduce the atmospheric dinitrogen to ammonia to the benefit of the plant [1], [2].

Rhizobial genomes are predicted to encode an unusually large repertoire of ATP-binding cassette (ABC) transporters dependent on a periplasmic solute binding protein (SBP), which guarantees bacteria to cope competitively with the oligotrophy of soil (e.g. 200 ABC genes in *S. meliloti* compared with 67 in *Escherichia coli*) [3], [4]. However, in nodules the classical model of nutrient cycling only involves the exchange of dicarboxylates and ammonium between the symbiotic partners [5].

Analyses of the regulons of the Sm-like RNA chaperone Hfq in a number of model bacterial species, including *S. meliloti* and its related plant pathogen *Agrobacterium tumefaciens*, have revealed a common massive misregulation of ABC transporter genes in the respective *hfq* mutants [6]–[12]. A large subset of Hfq-dependent ABC transporter mRNAs are direct targets of this protein, as revealed by deep sequencing-based surveys of Hfq-bound RNA conducted in some of these bacteria (e.g. *Salmonella* or *Rhodobacter sphaeroides*) [10], [13]. Hfq binds diverse RNA molecules, including regulatory small noncoding RNAs (sRNAs). Therefore, it has emerged as a global post-transcriptional regulator of gene expression with a great impact on bacterial physiology [14].

*Trans*-acting sRNAs are the largest and most intensively investigated group of functional untranslated RNA species identified in bacteria [15], [16]. These transcripts, ranging from 50 to 350 nucleotides (nt) in length, are differentially expressed in response to diverse environmental cues from intergenic regions (IGRs) of bacterial genomes [15]. Almost all the bacterial *trans*-sRNAs characterized to date act as post-transcriptional regulators of gene expression by an antisense mechanism that involves base pairing with single or multiple mRNA targets, thereby modulating their translation and/or stability. Riboregulation contributes to fine-tune a wide range of cellular processes such as general responses to abiotic stress, quorum sensing, virulence, or nutrient uptake and metabolism [15]–[17]. Remarkably, the activity of the *trans*-sRNAs commonly depends on Hfq in bacteria that express a recognizable homolog of this protein (i.e. almost half of all sequenced Gram-negative and Gram-positive species) [14], [18].

Recent systems-level surveys of the noncoding RNomes of *S. meliloti* and some related α-proteobacteria have delivered large sRNA catalogs that include hundreds of putative *trans*-acting riboregulators [19]. Only two of these *trans*-sRNAs termed AbcR1 and AbcR2, homologous to each other, have been functionally characterized to date, both in two model α-proteobacteria interacting with eukaryotic hosts; *A. tumefaciens* and the intracellular mammal pathogen *Brucella abortus* [20], [21]. Tandemly encoded homologs of these sRNAs had been previously identified by a series of genome-wide screens conducted in the reference *S. meliloti* strains Rm1021 and Rm2011, being indistinctly referred to as Smr15/16, Smr15C1/C2, Sra41, Sm3/3′ or SmelC411/SmelC412 [22]–[25]. For consistency they have been renamed here as their functionally characterized homologs, AbcR1 and AbcR2.

AbcR1/2 belong to the family of sRNAs designated αr15, which members always exist in multiple copies in the genomes of bacteria of the Rhizobiaceae and Brucellaceae families of the order Rhizobiales [26]. Homologous bacterial sRNA regulators can act either redundantly in a compensatory manner on the same pathways [27]–[29], additively each contributing to different extent to a single adaptive response [30], hierarchically upon each other in the same regulatory cascade [31] or independently, influencing on different or at most partially overlapping response pathways and target genes. In *A. tumefaciens* AbcR1 and AbcR2 sRNAs are co-regulated but have different targeting potential. AbcR1, but not AbcR2, silences three ABC transporter mRNAs, including the one encoding the periplasmic SBP of the plant-derived quorum sensing signal γ-amino butyric acid (GABA), thus predicting a function of this sRNA in phytopathogenesis [20]. Conversely, AbcR1 and AbcR2 act redundantly in *B. abortus* to regulate a set of uncharacterized amino acid and polyamine transporters, so that deletion of both sRNA *loci* is required to attenuate virulence [21]. Here, we provide evidences supporting an independent activity of the AbcR1 and AbcR2 sRNAs in *S. meliloti* Rm1021 that influences physiology of cultured bacteria but not the symbiotic interaction, most probably through the post-transcriptional regulation of different ABC uptake systems in an Hfq-dependent manner.

## Results

### The *S. meliloti* AbcR1 and AbcR2 sRNAs

**Figure 1:**
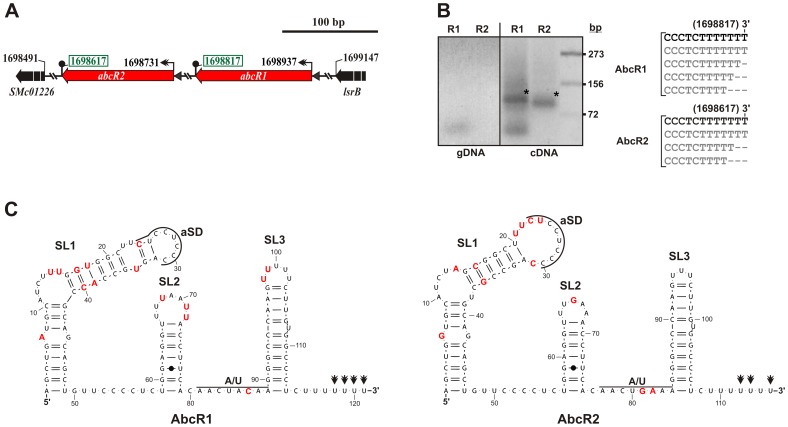
The *S. meliloti* AbcR1/2 sRNAs. A) Genomic region of the AbcR1 and AbcR2 sRNA *loci* in the chromosome of the reference strain Rm1021, indicating their flanking genes and relevant coordinates. Boxed numbers denote 3′-ends mapped in this work. B) Mapping of AbcR1/2 3′-ends. Electrophoretic separation (3% agarose gel) of PCR products from genomic DNA (gDNA; control reactions) and cDNA from 3′–5′ circularized wild-type RNA. Specific AbcR1/2 PCR products of the expected sizes cloned for sequencing are indicated with an asterisk (*). Band sizes (bp) of a co-migrating DNA marker are given to the right. The alignments of the AbcR1/2 3′-end regions inferred from different insert sequences are shown to the right of the panel. C) Predicted secondary structures of AbcR1 and AbcR2. Numberings denote relative nucleotide positions from the 5′-end of each molecule. Nucleotides representing differences between both sRNAs are indicated in red. Nucleotides complementary to the Shine-Dalgarno sequence (aSD) and potential Hfq-binding sites, i.e. A/U-rich region and terminal U residues determined by 3′-end mapping (double arrowheads) are indicated. SL, stem-loop domain.

Previous Northern hybridization experiments and 5′-RACE (Rapid Amplification of cDNA Ends) mapping revealed expression of AbcR1 and AbcR2 sRNAs from independent transcription units arranged in tandem between the chromosomal *SMc01226* and *lsrB* genes, which encode putative transcriptional regulators of the ArsR and LysR families, respectively, in strain Rm1021 (Fig. 1A). In this work, the 3′-ends of both sRNAs were experimentally determined by sequencing of the 3′/5′ junction fragments in cDNA obtained from circularized tobacco acid pyrophosphatase (TAP)-treated RNA (Fig. 1B). This analysis confirmed transcription initiation at the positions previously determined by RACE and mapped the 3′ boundaries at any of the last four residues of the oligo-U stretch of the predicted Rho-independent terminators, revealing no signs of polyadenylation in either of the two transcripts. The full-length AbcR1 and AbcR2 sRNAs are predicted to fold into similar secondary structures consisting of three hairpins (Fig. 1C). The 5′-loop of this structure exposes the conserved anti Shine-Dalgarno (aSD) sequence “UCCUCCC” that has been shown to mediate mRNA target recognition in the *A. tumefaciens* AbcR1 sRNA [20]. Both structures also evidence two signatures reported as preferred binding sites for Hfq [32]–[35], namely the A/U rich single-stranded region that precedes the Rho-independent terminator, which is well conserved in all αr15 relatives (Fig. S1), and the terminal U residues, predicted to remain unpaired in both sRNAs (Fig. 1C).

### AbcR1 and AbcR2 Exhibit Divergent Expression Profiles in *S. meliloti* Rm1021

**Figure 2:**
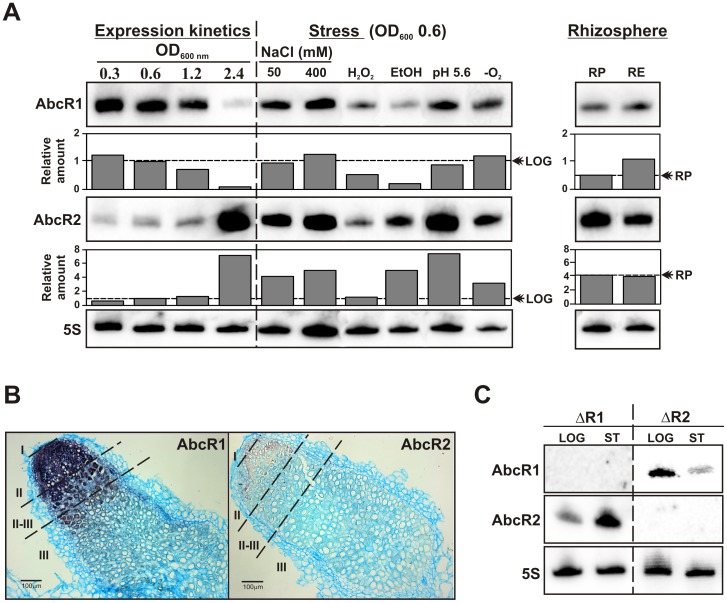
Divergent unlinked regulation of AbcR1 and AbcR2 in *S. meliloti* Rm1021. A) Northern blot detection of AbcR1 and AbcR2 transcripts in total RNA obtained at different OD_600_ (indicated above the panel) during Rm1021 growth in rich medium (expression kinetics), under different stresses and in rhizosphere-like conditions as indicated on top (see text for details). 5S rRNA probing (bottom panel) was used as RNA loading control. Plots underneath each blot correspond to the hybridization signal intensities normalized to the 5S RNA signal in each condition and the LOG expression (OD_600_ 0.6) of each sRNA. Values are given in arbitrary units. -O_2_, microoxic conditions; RP, Rigaud and Puppo medium; RE, root exudates (plant presence). The dotted line and the double arrowhead indicate the basal expression of AbcR1 and AbcR2 in untreated bacteria considered as the reference (LOG or RP) in each series of experiments. B) *In situ* hybridization of sections of *M. sativa* mature nodules occupied by Rm1021 with DIG-labeled riboprobes targeting AbcR1 and AbcR2. Zones of typical indeterminate nodules (I, II, II-III and III) are indicated. Bar represents 100 µm. C) Northern blot probing of RNA from exponential (LOG) and stationary phase (ST) cultures of Rm1021 deletion mutant derivatives ΔR1 and ΔR2 for detection of AbcR1 and AbcR2 as indicated to the left. 5S probing of the same RNA samples is shown in the bottom panel.

Probing of RNA obtained under a limited number of biological conditions anticipated that the AbcR1 and AbcR2 sRNAs are differentially expressed in cultured and endosymbiotic *S. meliloti* Rm1021 bacteria [22]. In this work we have investigated the expression profiles of these sRNAs under a broader range of stress conditions and during symbiosis of Rm1021 with alfalfa plants (Fig. 2A; Fig. 2B). Despite their high sequence identity, AbcR1 and AbcR2 species were specifically detected on Northern blots of RNA extracted from cultured bacteria with 25-mer oligonucleotide probes targeting the variable 5′ region of each transcript (Fig. 1C; Fig. S1).

Expression kinetics of these sRNAs during Rm1021 growth in complete rich medium (TY) revealed that AbcR1 was highly expressed in exponentially growing bacteria whereas it was barely detected upon entry of the culture into stationary phase. Conversely, AbcR2 progressively accumulated to reach its maximum levels in stationary phase (Fig. 2A). We next examined AbcR1 and AbcR2 expression in stressed bacteria (see Materials and Methods for further details about culture conditions). Considering the levels of each transcript in log cultures as the reference, AbcR1 abundance remained unaltered or even decreased under certain stresses (e.g. oxidative and EtOH-induced membrane stress) whereas up-regulation of AbcR2 was observed upon osmotic upshift (∼4–5 fold), membrane stress (∼5-fold), moderate acidity (∼7-fold) and microaerobiosis (∼3-fold) (Fig. 2A).

To assess AbcR1 and AbcR2 expression at early symbiotic stages bacteria cultured in TY broth to exponential phase were pelleted and resuspended in nitrogen-free mineral solution (i.e. Rigaud and Puppo; R&P) to inoculate alfalfa plants grown hydroponically in the same medium. Total RNA was then obtained 20 h after plants inoculation to probe AbcR1 and AbcR2 expression under the influence of the root exudates (RE). As the reference in these experiments RNA preparations from bacteria incubated during the same period of time in the R&P solution in the absence of the plant were also probed (Fig. 2A, right panel). It should be noted that the R&P solution enables rhizobial survival but does not promote growth (i.e. it is devoid of any nutrient source), which requires the presence of the plant. Northern blot hybridizations revealed an increase (∼2-fold) in the levels of AbcR1 when bacteria were incubated in the presence of alfalfa, probably in correlation with the modest growth rates supported by the RE. In contrast, the expression pattern of AbcR2 resembled that of this transcript in stationary phase bacteria and was not influenced by the plant. Therefore, nutrient deprivation imposed by the plant mineral solution was likely the environmental factor that induced expression of AbcR2 in these assays.

To analyze AbcR1 and AbcR2 expression in endosymbiotic bacteria a series of longitudinal sections of 30 days-old nodules collected in the course of the plant assays were hybridized under high stringent conditions with digoxigenin (DIG)-labeled riboprobes from both transcripts (Fig. 2B). Sense probes and a riboprobe targeting the plant carbonic anhydrase mRNA known to be highly abundant in alfalfa nodule tissues were used as negative and positive controls of the *in situ* hybridizations, respectively (data not shown). A strong hybridization signal corresponding to AbcR1 was detected in the so-called invasion zone II of the nodules, which is occupied by branched infection threads containing vegetative undifferentiated dividing bacteria. The intensity of the signal decreased throughout the interzone II-III, where bacteroid differentiation begins, and became undetectable in plant cells hosting mature nitrogen-fixing bacteroids (zone III). In contrast, only a rather faint expression of AbcR2 was detected in zone II of the nodule tissues.

Finally, a new series of Northern hybridization experiments revealed that AbcR1 retained its growth-dependent accumulation profile in TY broth in a Rm1021 AbcR2 deletion mutant derivative and vice versa, suggesting that the expression of these sRNAs does not depend upon each other (Fig. 2C).

Altogether, these findings indicate that transcription of AbcR1 and AbcR2 in Rm1021 only occurs in free-living and undifferentiated symbiotic bacteria and is divergently regulated.

### Lack of Hfq Compromises AbcR1 and AbcR2 Stability

**Figure 3:**
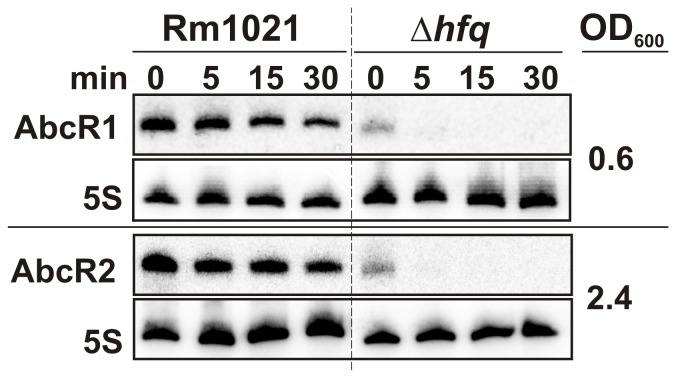
Hfq contributes to stabilize AbcR1/2 in Rm1021. Northern blot analysis of AbcR1 and AbcR2 decay in Rm1021 and its *hfq* deletion mutant derivative (Δ*hfq*) upon transcription arrest with rifampicin. Bacteria were grown to both exponential (OD_600_ 0.6) and stationary (OD_600_ 2.4) phases in TY broth and samples were withdrawn prior to or at the time-points (in min) indicated above the panels after antibiotic addition.

In agreement with the *in silico* predictions both AbcR1 and AbcR2 sRNAs have been previously shown to co-inmunoprecipitate with a chromosomally-encoded FLAG epitope-tagged Hfq protein in *S. meliloti* Rm1021 [9]. Rifampicin-treatment experiments were therefore conducted to assess the Hfq-dependent turnover patterns of these transcripts. RNA samples extracted from log (OD_600_ 0.6) and stationary (OD_600_ 2.4) phase cultures of Rm1021 and its *hfq* deletion mutant derivative (1021Δ*hfq*; [9]) before or at 5, 15 and 30 min upon transcription arrest were probed to detect AbcR1 (RNA from log cultures) and AbcR2 (RNA from stationary cultures) (Fig. 3). In the wild-type background the estimated half-life was 33 min for AbcR1 and 18 min for AbcR2. In the absence of Hfq accumulation of both transcripts was visibly reduced as compared to their wild-type levels even before transcription arrest (t = 0). Only 5 min after rifampicin addition both sRNAs were hardly detectable, rendering half-life determination impossible at this time scale. These results further support that AbcR1 and AbcR2 are Hfq-dependent sRNAs.

### Growth and Symbiotic Phenotypes of *S. meliloti* AbcR1 and AbcR2 Mutants

**Figure 4:**
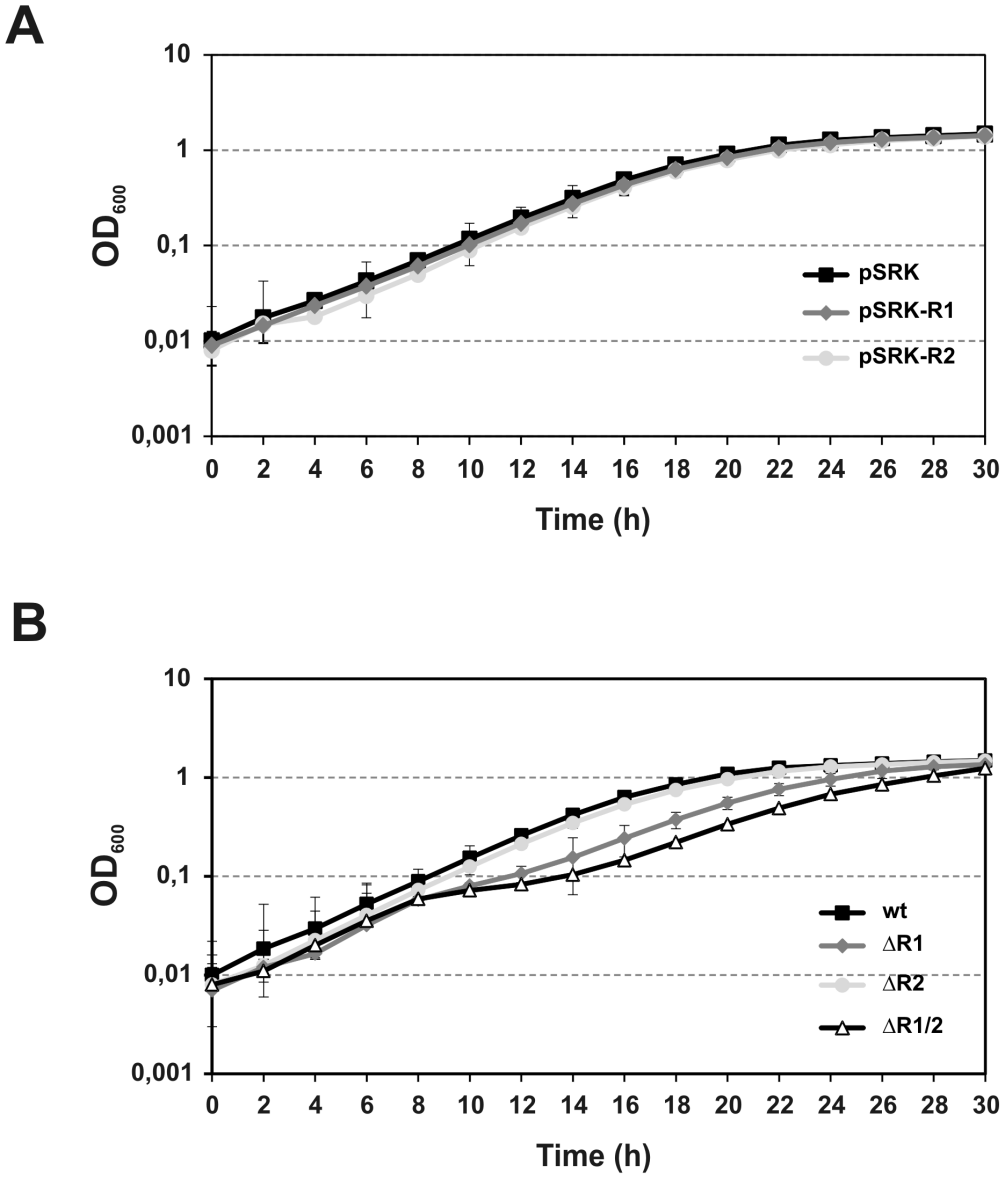
Growth phenotype of the Rm1021 AbcR1/2 mutants. Growth curves in TY broth of Rm1021 harboring pSRK-R1, pSRK-R2 or the empty control vector pSRK (A) and the wild-type Rm1021 and its AbcR1/2 deletion (ΔR1, ΔR2 and ΔR1/2) mutant derivatives (B). OD_600_ readings were determined in triplicate at 2 h intervals from two independent cultures of the tested strains. Standard errors bars are indicated.

As a first approach to address the biological function of AbcR1 and AbcR2 sRNAs, we assessed the growth and symbiotic phenotypes of *S. meliloti* Rm1021 single (ΔR1 and ΔR2) and double (ΔR1/2) deletion mutants as well as of derivatives constitutively (over)expressing each sRNA independently. The latter series of mutant strains was obtained by mobilization to Rm1021 of mid-copy plasmids (∼30–40 copies/cell) expressing AbcR1 or AbcR2 (pSRK-R1 and pSRK-R2, respectively) from an engineered constitutive P_lac_ promoter (Fig. S2). Growth kinetics of the Rm1021 derivatives harboring pSRK (empty control plasmid), pSRK-R1 or pSRK-R2 was identical (Fig. 4A). Similarly, growth curves in TY broth did not reveal differences between the behavior of the wild-type strain (generation time, *g* = 3.49 h) and that of mutant ΔR2 (*g* = 3.54 h). In contrast, both ΔR1 and ΔR1/2 mutants showed comparable slightly delayed log phase (*g*-values of 3.79 and 3.91 h, respectively) but, nonetheless, reached the stationary phase at similar optical density than the parent strain (Fig. 4B). These results indicate that only the activity of AbcR1 has some impact on *S. meliloti* physiology during exponential growth of bacteria in rich medium.

As symbiotic tests, we conducted nodulation competitiveness assays in which alfalfa plants grown hydroponically in tests tubes were co-inoculated with mixtures of two bacterial strains, each containing a GUS-tagged Rm1021 derivative (marker strain) [36] and either of the AbcR1/AbcR2 mutants (deletion and overexpression series) at different ratio (1∶1, 10∶1 and 1∶10) (Fig. S3). There was a correlation between the percentages of nodule occupancy of each strain in the assays, as inferred from the number of blue (GUS-tagged bacteria) and white (tested strains) nodules, and their representation in the co-inoculation mixtures. However, statistical tests did not support significative differences between the nodulation competitiveness rates of the reference strains (i.e. Rm1021 or 1021pSRK) and those of the mutant derivatives. Furthermore, mature nodules occupied by either of the mutants showed no obvious histological alterations and were of pink color, evidencing plant leghemoglobin expression and active symbiotic nitrogen-fixation (data not shown). We therefore conclude that neither AbcR1 nor AbcR2 are required for the competitive and efficient nodulation of *S. meliloti* on alfalfa roots under laboratory conditions.

### AbcR1 Down-regulates the Periplasmic SBP LivK

**Figure 5:**
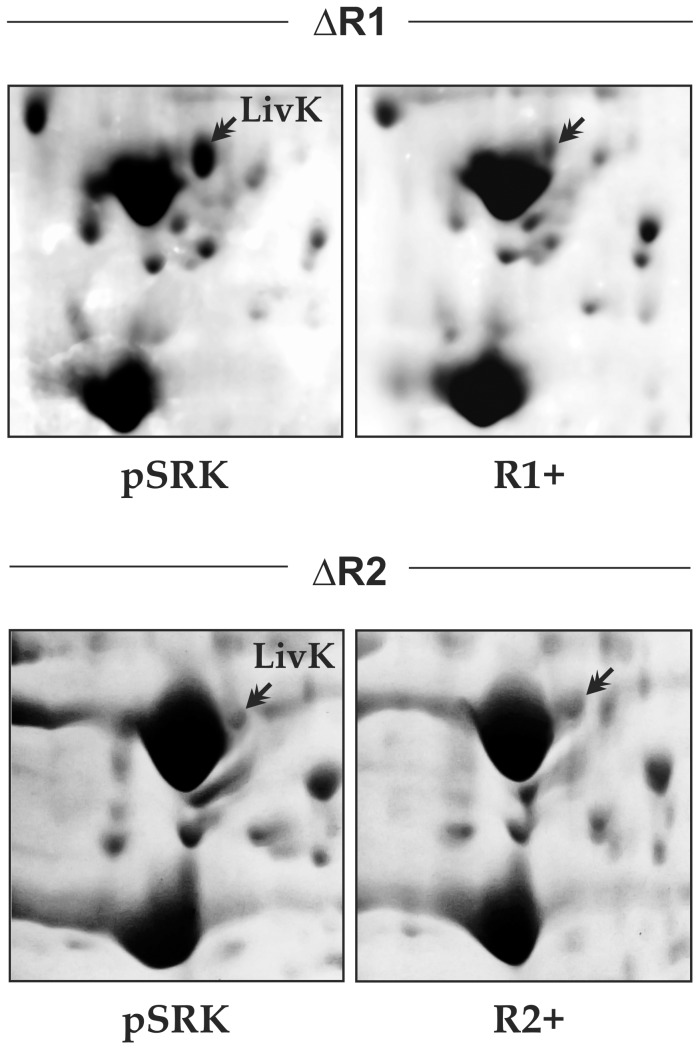
AbcR1 down-regulates the periplasmic protein LivK. 2D-PAGE analysis of periplasmic protein extracts from the Rm1021 ΔR1 and ΔR2 mutants (as indicated above the panels) carrying the control plasmid pSRK or derivatives (over)expressing AbcR1 (R1+) or AbcR2 (R2+), all grown in TY broth to exponential phase. Relevant subsections of representative Coomassie-stained 2D gels are shown. Arrows indicate the LivK protein spots.

The *A. tumefaciens* and *B. abortus* AbcR1 and AbcR2 homologs have been shown to target a handful of mRNAs encoding the periplasmic components of ABC transporters. This evidence prompted us to analyze the AbcR1- and AbcR2-dependent Rm1021 periplasmic proteome. Specifically, the periplasmic protein fractions of ΔR1 and ΔR2 mutants carrying the control plasmid pSRK or the mid-copy plasmids pSRK-R1 (R1+) or pSRK-R2 (R2+), all grown to log phase (OD_600_ 0.6) in TY, were resolved on two-dimensional gels (Fig. 5). Analysis of at least four series of Coomassie-stained 2D gels revealed, among other minor unreliable differences, a consistent up-regulation of one protein in bacteria lacking AbcR1 (ΔR1pSRK) (Fig. 5, upper left panel). Mass spectrometry (MALDI-TOF) identified this differentially accumulated polypeptide as LivK (MW 39 kDa and pI 5.0), which is encoded by the chromosomal *SMc01946* gene. LivK has been shown to mediate branched-chain amino acid (leucine, isoleucine and valine; LIV) uptake in *S. meliloti* [37] and is homologous to the *A. tumefaciens* Atu2422 periplasmic SBP of the ABC GABA transporter that has been identified as target of AbcR1 in this bacterium [20].

Reduced LivK levels in the ΔR1 mutant complemented with pSRK-R1 (Fig. 5A, upper right panel) were comparable to those observed in the absence of AbcR2 (ΔR2pSRK; Fig. 5, bottom left panel). This is probably because of silencing by AbcR1, which is actively transcribed from its chromosomal *loci* in a ΔR2 background during exponential growth (Fig. 2C). Nonetheless, expression of AbcR2 from pSRK-R2 did not alter LivK accumulation in ΔR2 (Fig. 5, bottom right panel), suggesting that this sRNA does not contribute to the regulation of this protein.

### A Double-plasmid Reporter Assay Confirmed Targeting of *livK* by AbcR1 *in vivo*

**Figure 6:**
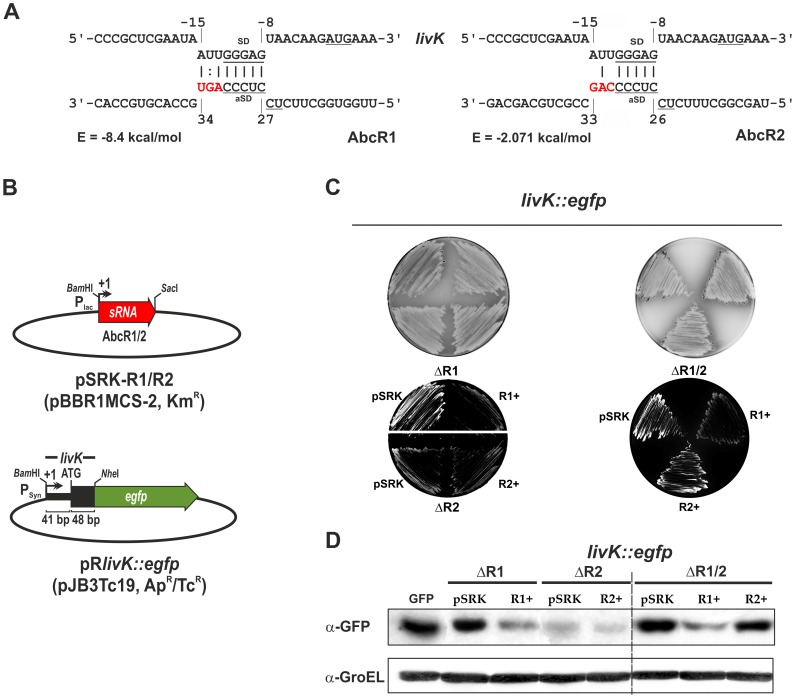
Post-transcriptional regulation of the *livK* mRNA by AbcR1. A) IntaRNA predicted interaction between the *livK* mRNA and the AbcR1 (left) and AbcR2 (right) transcripts. The anti Shine-Dalgarno (aSD) motif of the sRNAs as well as the Shine-Dalgarno sequence and AUG start codon of the *livK* mRNA are underlined. Numberings denote positions relative to the AUG start codon of the mRNA and the transcription start site of the sRNAs. The predicted minimum hybridization energy (E) is indicated in each case. Nucleotides in red denote mismatches between the AbcR1 and AbcR2 sequences complementary to the *livK* mRNA. B) Diagrams of the compatible broad host-range pBBR1MCS-2 (mid-copy) and pJB3Tc19 (low-copy) plasmid derivative constructs expressing the AbcR1/2 sRNAs and the translational *livK::egfp* fusion, respectively. sRNA genes were cloned as *Bam*HI-*Sac*I fragments under the control of the constitutive version of the P_lac_ promoter and the 5′ region of the *livK* mRNA (5′-UTR and the first 16 codons) was inserted as a *Bam*HI-*Nhe*I fragment right downstream of a constitutive P_syn_ promoter so that transcription of both the sRNA and the fusion mRNA precisely starts from their native +1 sites in pSRK-R1/pSRK-R2 and pR*livK::egfp*, respectively. C) Agar plate-based colony fluorescence of the reporter Rm1021 ΔR1 and ΔR2 single mutants (left) and ΔR1/2 double mutant (right) co-transformed with the fusion vector pR*livK::egfp* and plasmids pSRK (control vector), pSRK-R1 (R1+) or pSRK-R2 (R2+) as indicated in the panels. Images of the same plates in the visible light are also shown on top. D) Western blot probing for detection of the LivK::EGFP fusion protein in total protein extracts from reporter strains containing the same plasmid combinations as in C (indicated on top of the panel) and grown in TY broth to exponential phase (OD_600_ 0.6). As positive control, total protein extracts from an Rm1021 derivative carrying plasmid pJB_EGFP, which constitutively expresses EGFP, were also probed with the same antibody. GroEL was probed as protein loading control.

The full-length *livK* mRNA sequence was scanned for antisense interactions with AbcR1 and AbcR2 using the program IntaRNA. As searching parameters we imposed a minimum interaction seed of seven paired bases and at most one unpaired base with a full weighting of the interaction site accessibility [38], [39]. IntaRNA predicted a unique interaction site of such characteristics between AbcR1 and the *livK* message involving a short sequence stretch of 8 nt (positions 27 to 34 in the sRNA and −8 to −15 relative to the A residue of the AUG start codon in the mRNA) that includes the conserved aSD sequence within the 5′ hairpin loop of the sRNA (5 nt) and the ribosome binding site (RBS) of the mRNA, with estimated hybridization energy of −8.4 kcal/mol (Fig. 6A, left diagram). The same analysis did not reveal any antisense interaction between AbcR2 and *livK* fulfilling the searching criteria. The complementarity between AbcR2 and the 8-nt stretch predicted as target of AbcR1 within the 5′ untranslated region (UTR) of the *livK* mRNA is reduced to six nucleotides with only five consecutive in both molecules (aSD-RBS; hybridization energy −2.1 kcal/mol) (Fig. 6A; right diagram).

To further assess *livK* mRNA regulation by AbcR1 we used an adapted version of the reporter system previously developed to study riboregulation in enterobacteria [40] (Fig. 6B). A *S. meliloti* Rm1021 genomic region spanning the entire 5′-UTR (41 nt) and the first 16 codons of the *livK* mRNA was translationally fused to the N-terminus of EGFP and placed under the control of the constitutive P_syn_ promoter [41] in the low-copy pJB3Tc19-derived plasmid pR_EGFP, so that the cloning strategy ensured transcription of the fusion from the native +1 site of *livK* (see Materials and Methods for details on the plasmid construct).

In a first series of experiments, Rm1021 ΔR1 and ΔR2 single mutants were co-transformed with the fusion plasmid (pR*livK::egfp*) and either of the compatible plasmids pSRK-R1, pSRK-R2, or the control vector pSRK. Double transconjugants carrying the different plasmid combinations were first checked for colony fluorescence on TY agar plates that were visually inspected (Fig. 6C, left panels). The highest fluorescence was evident in the absence of AbcR1 (i.e. in ΔR1pSRK), whereas the expression of this sRNA from either pSRK-R1 in ΔR1 (R1+) or the chromosome in ΔR2pSRK visibly reduced fluorescence of the *livK::egfp* fusion. In contrast, neither the endogenous nor ectopic (i.e. from pSRK-R2) expression of AbcR2 in the ΔR1 and ΔR2 strains, respectively, influenced the visible AbcR1-dependent fluorescence. Therefore, the fluorescence intensity of this set of reporter strains fully correlates with the accumulation profiles of the chromosomally encoded LivK protein in the same AbcR1/2 genetic backgrounds (Fig. 5). To circumvent any influence of the endogenous expression of AbcR1 and AbcR2 on the fluorescence of the *livK::egfp* fusion, pSRK, pSRK-R1 and pSRK-R2 were independently mobilized to the Rm1021 ΔR1/2 double mutant derivative carrying pR*livK::egfp* (Fig. 6C, right panel). The visible fluorescence pattern of this new set of reporter strains unequivocally demonstrated regulation of *livK* by AbcR1 but not by AbcR2. Finally, Western-blot probing of protein extracts from the whole set of *S. meliloti* Rm1021 reporter strains grown to log phase in TY broth to detect the LivK::EGFP fusion protein fully confirmed the colony fluorescence observations (Fig. 6D).

These results suggest that the AbcR1-mediated control of LivK is exerted by a canonical antisense interaction between the sRNA and the 5′-UTR of the mRNA, which probably results in occlusion of the RBS and translational inhibition. The productive base-pairing is predicted to involve a sequence stretch within the 5′ hairpin of AbcR1, which contains an aSD motif and differs in three nucleotides respect to its equivalent region in AbcR2.

## Discussion

In the present study, we have addressed the functional characterization of the *S. meliloti* homologous *trans*-acting AbcR1 and AbcR2 sRNAs. Multiple sRNA copies are not unusual in bacteria but the physiological/ecological advantages of these reiterations are scarcely understood [15], [26], [42]. Their divergent unrelated expression profiles, the exclusive contribution of AbcR1 to a growth phenotype and their specific targeting potential are evidences of independent regulatory functions of these sRNAs in this bacterium. The latter is exemplified by the contribution of AbcR1, but not of AbcR2, to the post-transcriptional silencing of the *livK* mRNA. To the best of our knowledge this is the first *trans* sRNA-mRNA target pair identified in a legume symbiont.

AbcR2 and AbcR1 display divergent accumulation kinetics during growth of *S. meliloti* Rm1021 in rich broth, in contrast to their *A. tumefaciens* counterparts, which are both induced simultaneously upon entry of bacteria into stationary phase [20]. Remarkably, these growth-dependent expression profiles were found to be unlinked (i.e. the absence of one sRNA did not influence the amount of the other), indicating that AbcR1 and AbcR2 do not act in a hierarchical manner in *S. meliloti*. A great proportion of the bacterial sRNAs characterized so far exhibit stationary phase expression as does AbcR2, which anticipates transcriptional control by diverse stress regulons progressively induced upon nutrient starvation and cessation of growth [43]. In agreement with this assumption, salinity, acidity, membrane stress, microaerobiosis and nutrient deprivation in the plant growth media were mimicked environmental conditions that stimulated AbcR2 expression. Some of the environmental signals that favored AbcR2 accumulation are also known to contribute to govern symbiotic gene expression *in planta* (e.g. oxidative burst, microaerobiosis or intracellular acidity). However, no signs of expression of this transcript were found in mature nodules, further evidencing the complexity of the signaling between the partners in symbiosis [44]. In contrast, AbcR1 transcription was induced in log cultures, by the root exudates in the rhizosphere and in the invasion zone of the nodules occupied by undifferentiated bacteria. This expression pattern resembles that of rhizobial genes involved in the utilization of plant-derived nutrient sources such as *mio*-inositol, α-glucosides or proline [45]–[48]. Therefore, AbcR1 seems to operate under plentiful nutrient conditions in actively dividing bacteria rather than under stress. Conserved motifs in the promoter regions of AbcR1 and AbcR2 further support their transcriptional control by likely unrelated yet uncharacterized transcription factors [26].

There is increasing evidence that the sRNA regulators primarily act to fine-tune stress responses that commonly rely on redundant bacterial pathways [15]. Consequently, end-point assays to assess physiological phenotypes of deletion strains usually fail to evidence sRNA function. We have showed that only deletion of the AbcR1 *loci* resulted into a discrete but nonetheless reliable growth delay of Rm1021 in culture but both sRNAs, AbcR1 and AbcR2, are dispensable for the establishment of a wild-type symbiosis. Interestingly, the *B. abortus* AbcR1 and AbcR2 sRNAs have been shown to fulfill redundant functions that influence short-term bacterial survival within murine macrophages and chronic spleen colonization [21]. It is well known that rhizobial and brucellae species have retained similar genes and common strategies for the establishment of chronic intracellular infections in widely diverse eukaryotic hosts [49]. Therefore, our results evidence a rather different impact of AbcR1 and AbcR2 activity in the biology of *S. meliloti* and *B. abortus*.

As bacterial *trans*-acting sRNAs, the identity of the AbcR1 and AbcR2 mRNA targets is likely the most relevant information to pinpoint their cellular functions. *Trans*-encoded antisense sRNAs typically display short, discontinuous and imperfect complementarity to their targets. Consequently, target identification is a big challenge usually relying on computational predictions and further experimental validation [50]. Proteomics identified the *livK* mRNA, encoding a *S. meliloti* LIV periplasmic SBP, as target of AbcR1. LivK has broad substrate specificity in *S. meliloti*, which genome encodes at least other SBP (AapJ) with similar uptake ability [37]. AapJ has been neither revealed by our proteome analysis nor predicted *in silico* to be targeted by AbcR1/2 [11]. A *S. meliloti* LivK/AapJ double deletion mutant is not impaired for symbiosis with alfalfa [37], which adds an explanation to the lack of symbiotic phenotypes of the AbcR1/2 mutants.

Similar approaches also identified several ABC transporter mRNAs as AbcR1 and AbcR2 targets in *A. tumefaciens* and *B. abortus* [20], [21]. Preliminary computational predictions have rendered long and partially overlapping lists of target candidates for AbcR1 and AbcR2 in *S. meliloti*, with 35–45% of the top scoring hits corresponding to ABC transporter genes [11]. Altogether these findings suggest that the members of the αr15 family of sRNAs regulate multiple ABC transport systems in individual α-proteobacteria, thus resembling the function of GcvB in enterobacteria [51]–[53]. Therefore, *livK* mRNA targeting probably does not reflect the full regulatory potential of AbcR1/2 sRNAs in *S. meliloti*. Further work based on more sensitive quantitative high-throughput approaches conducted in biological conditions that induce AbcR1/2 expression will be required to characterize the complete AbcR1/2 target repertoire and corroborate this hypothesis.

Some of the AbcR1/2 mRNA target interactions have been confirmed either *in vitro* [20] or *in vivo* in the heterologous host *E. coli* [21] using assays that override any contribution of host factors (e.g. Hfq or other unknown factor) to the regulatory activity of the sRNAs. We have further assessed targeting of *livK* by AbcR1/2 in their natural host, *S. meliloti* Rm1021, using a double-plasmid reporter assay that uncouples transcriptional regulation of both the sRNA and its putative mRNA target from chromosomal control, as described for enterobacteria [40]. Therefore, our broad host-range sRNA expression and target reporter vectors are reliable tools for the analysis of the sRNA-mediated translational control and target recognition in any compatible α-proteobacteria. This assay confirmed *livK* mRNA regulation by AbcR1 but not by AbcR2, further supporting an independent regulatory potential of these sRNAs, similar to that reported for AbcR1 and AbcR2 in *A. tumefaciens* [20]. In contrast, although direct regulation of *livK* by AbcR1 and AbcR2 in *B. abortus* has not been tested, a similar reporter assay in the *E. coli* genetic background revealed redundant activity of these sRNAs in the regulation of at least three mRNA targets [21].

Several observations indicate that AbcR1 and Hfq act in concert to inhibit translation of the *livK* mRNA and to accelerate its decay. First, AbcR1 is predicted to bind the *livK* mRNA at the SD sequence. This would interfere with translation initiation as suggested by the reporter assay *in vivo* and demonstrated by toeprinting mapping of the interaction of the AbcR1 sRNA with the *livK* mRNA homolog (*atu2422*) in *A. tumefaciens* [20]. Second, AbcR1/2 sRNAs are transcribed as non polyadenylated highly stable RNA species which rapidly decay in the absence of Hfq in different *S. meliloti* strains as revealed by our assays and results reported by others [54]. Accordingly, proteome and transcriptome analyses have revealed up-regulation of *livK* in a *S. meliloti hfq* deletion mutant [7]–[9], [11]. Finally, the AbcR1/2 sRNAs and the *livK* mRNA bind Hfq as revealed by Northern [9] and deep-sequencing analysis (J.I. Jiménez-Zurdo and A. Becker, unpublished) of transcripts co-inmunoprecipitated with a tagged version of the chaperone.

Most of the predicted interactions of AbcR1 and AbcR2 with their targets involve nucleotides that remain largely unpaired within the first hairpin of both molecules. Despite sharing an aSD motif, this short sequence stretch (∼12–14 nt) is variable in most of the chromosomal αr15 sRNA duplicates encoded by α-proteobacteria but not in the brucellae AbcR1/2 sRNAs that retained a conserved 5′ loop [26]. Therefore, it is tempting to speculate on the 5′-hairpin of the αr15 sRNA pairs as the functional discriminatory domain for the targeting of specific sets of mRNAs in each individual bacterial species [26], [55].

To summarize, our findings suggest that the *S. meliloti* AbcR1 and AbcR2 sRNAs each responds to different environmental cues to optimize the uptake of available nutrients in free-living and undifferentiated nodule invading rhizobia, most probably by the post-transcriptional regulation of largely independent sets of ABC transport systems in an Hfq-dependent manner. Our work therefore highlights the rather different outcomes of the activity of redundant homologous *trans*-acting sRNAs in phylogenetically related bacterial species.

## Materials and Methods

### Bacterial Strains, Plasmids and Culture Conditions

Bacterial strains and plasmids used in this study along with their relevant characteristics are listed in Table S1. *S. meliloti* wild-type Rm1021 and mutant derivative strains were routinely grown in complex tryptone-yeast TY medium [56] at 30°C. *E. coli* strains were grown in Luria-Bertani (LB) medium at 37°C. Antibiotics were added to the media when required at the following final concentrations (µg/ml): streptomycin (Sm) 250, ampicillin (Ap) 200, tetracycline (Tc) 10, erythromycin (Er) 100, and kanamycin (Km) 50 for *E. coli* and 180 for rhizobia.

Bacteria in exponential and stationary growth phases were obtained by incubation of TY cultures to OD_600_ 0.6 and 2.4, respectively. For stress induction in TY broth, the medium and growth conditions were modified as follows. Moderate salinity and membrane stress were mimicked by supplementing the medium with 50 mM NaCl and 2% v/v EtOH [57], respectively, and growth of bacteria to OD_600_ 0.6. The osmotic upshift and oxidative stress were imposed by adding 400 mM NaCl and 1 mM H_2_O_2_, respectively, to exponentially growing bacteria and further 1 h incubation of the cultures. Acidic stress was generated by re-suspension of bacteria (grown to OD_600_ 0.6) in TY medium buffered at pH 5.6 with 20 mM MES followed by 1 h incubation. Finally, microaerobiosis was recreated by flushing log TY cultures with a 2% oxygen-98% argon gas mixture during 10 min and further 4 h incubation of bacteria in this condition.

To assess Hfq-dependent AbcR1 and AbcR2 decay Rm1021 and its Δ*hfq* derivative [9] were grown in 150 ml of TY broth until exponential and stationary phase, and transcription was terminated by rifampicin addition at final concentration of 800 µg/ml. Aliquots (10 ml) of the cultures were withdrawn immediately before rifampicin addition and at time-points after the arrest of transcription (5, 15 and 30 min) for RNA extraction.

When appropriate growth rates of rhizobial strains in TY broth were monitored in an automated BioScreen C MBR machine (Growth Curves USA, Piscataway, NJ) as described [9].

### DNA Oligonucleotides

Sequences of all the oligonucleotides used for cloning and as probes in Northern hybridization experiments are provided in Table S2.

### Plant Growth and Inoculation

*Medicago sativa* L. ‘Aragón’ (alfalfa) seeds were surface sterilized and germinated as described [58]. Groups of ten seedlings were placed on meshes made of aluminum paper on 50-ml Falcon tubes as sterile plant containers previously filled with 40 ml of nitrogen-free mineral solution (Rigaud and Puppo; R&P) [59] and maintained there under controlled light and temperature conditions (16 h light at 24–26°C and 8 h dark at 20–22°C). For the preparation of the inoculum, bacteria (*S. meliloti* Rm1021) grown to log phase in TY broth were centrifuged, washed and re-suspended in the R&P solution. Seven days-old plantlets were inoculated with the rhizobial suspensions at a final concentration in the plant growth medium of 10^6^ cells/ml. Total RNA was extracted from bacteria 20 h after plants inoculation.

Alternatively, for the collection of mature nodules, individual alfalfa seedlings were cultured in test tubes and inoculated with Rm1021 as described [58].

### RNA Preparation and Northern Analysis

Total RNA was isolated from bacteria subjected to all the described culture conditions by acid phenol/chloroform extraction as reported previously [60]. RNA samples (10 µg) were separated on 6% polyacrylamide/7 M urea gels, blotted onto nylon membranes and probed with 5′-end radiolabeled 25-mer oligonucleotides specific for the AbcR1/2 sRNAs as described [22]. Hybridization signal intensities were quantified with the Quantity One software package (Bio-Rad).

### Determination of 3′-ends

Experimental determination of the 3′-ends of the AbcR1 and AbcR2 transcripts was carried out according to [31]. Briefly, total RNA was extracted from TY cultures of *S. meliloti* Rm1021 grown to OD_600_ 0.6 (log phase) and 2.4 (stationary phase) and subsequently treated (8 µg) with 10 U of tobacco acid pyrophosphatase (TAP; Epicentre) during 10 min at 37°C, followed by organic extraction. TAP-treated RNA was circularized with 40 U of T4 RNA ligase at 17°C (New England Biolabs) in overnight reactions. Following organic extraction and ethanol precipitation, 2 µg of self-ligated RNA were reverse-transcribed with 200 U of SuperScript™ II (Invitrogen) using random hexamers (50 ng) as primers in a 20-µl reaction. After annealing (70°C, 10 min), reactions were incubated 2 h at 42°C before addition of the enzyme. Upon enzyme inactivation (85°C, 5 min), samples were treated with 1 U of RNase H (Roche) at 37°C for 20 min. A total of 1 µl of the cDNA preparation was used as template in standard PCR reactions using a *Taq* polymerase and primer pairs (5–15C1cir/3–15C1cir and 5–15C2cir/3–15C2cir) designed to amplify fragments containing the 3′/5′ junctions of each sRNA. PCR products were separated in TAE 3% agarose gels and cloned into pGEM®-T easy (Promega) for sequencing.

### *In situ* Hybridization of Nodule Tissues

*M. sativa* 30 days-old nodules infected with *S. meliloti* Rm1021 were processed and subjected to *in situ* hybridization under high stringent conditions as described [61]. DIG-labeled AbcR1 and AbcR2 sense (negative controls) and antisense riboprobes were generated by *in vitro* transcription using as templates DNA fragments obtained by PCR amplification of plasmids pKS-R1 and pKS-R2 (Table S1) with the primer pair M13_F/M13_R (Life Technologies). As a positive control, nodule sections were hybridized with a probe specific for the *M. truncatula* carbonic anhydrase [62].

### Construction of the *S. meliloti* AbcR1/2 Mutants

*S. meliloti* Rm1021 ΔR1, ΔR2 and ΔR1/2 mutant strains were constructed by replacement of the chromosomal sRNA *loci* by a 135-bp Er resistance cassette (SSDUT1) designed in our laboratory. First, a 2,210-bp DNA region containing the AbcR2 and AbcR1 *loci* and flanking sequences (1,143-bp downstream of the 3′-end of AbcR2 and 1,061-bp upstream of the 5′-end of AbcR1) was PCR amplified using Rm1021 genomic DNA as the template and the primer pair 5–15C1/3–15C2, which adds the restriction sites *Xba*I and *Sph*I to the 5′- and 3′-ends of the amplicon, respectively. The resulting fragment was inserted into the pGEM-T Easy vector (Promega) yielding pGEMgR1/2. This plasmid was amplified with the pairs of divergent primers 5–15C1-i/3–15C1-i, 5–15C2-i/3–15C2-i, and 5–15C1-i/3–15C2-i all carrying an internal *Kpn*I restriction site and flanking the coding sequences of AbcR2, AbcR1 and the AbcR1/2 tandem, respectively. The resulting PCR products were digested with *Kpn*I and self-ligated to generate plasmids pGEMΔR2, pGEMΔR1 and pGEMΔR1/2 containing the single AbcR2 and AbcR1 and the double AbcR1/2 deletions, respectively.

The SSDUT1 cassette consists of the constitutive P_syn_ promoter (S) [41], the coding sequence of the pentapeptide that confers Er resistance [63], [64] preceeded by an optimal ribosome binding site (SDU) and the rrnB T1 transcriptional terminator (T1) [65]. It was constructed as follows. The P_syn_ promoter was amplified from plasmid pBBSyn [66] with the primers SalSyn and SynXho that incorporate the *Sal*I and *Xho*I sites to the 5′- and 3′-ends of the fragment, respectively. This PCR fragment was inserted into pGEM-T Easy to generate pGEMSSynX. The SDU element was generated by annealing of the 47-mer oligonucleotides fwSDU-p and rvSDU-p which were designed to leave 5′-end overhangs complementary to *Sal*I and *Xho*I recognition sites, and inserted into the *Xho*I site of pGEMSSynX, yielding pGEMSSDUX. The transcriptional terminator (T1) was obtained by PCR amplification of plasmid pICT1 [65] with primers 5′T1 and 3′T1 that add *Sal*I and *Xho*I sites, respectively, to the fragment, which was inserted into pGEM-T Easy to obtain pGEMT-T1. The SSDU elements of the cassette were retrieved from pGEMSSDUX as a single *Sal*I-*Xho*I fragment that was inserted into the *Sal*I site preceding the T1 terminator in pGEMT-T1 to yield pGEMSSDUT1. The full-length cassette was finally amplified from the latter plasmid with the primer pair 5-Ery-Kpn/3-Ery-Kpn that incorporates a *Kpn*I restriction site to both ends of the fragment.

The SSDUT1 cassette was then inserted into the unique *Kpn*I site of pGEMΔR1, pGEMΔR2 and pGEMΔR1/2 generating pGEM-EryΔR1, pGEM-EryΔR2 and pGEM-EryΔR1/2. The inserts of these plasmids were recovered by *Xba*I-*Sph*I restriction and ligated to the suicide vector pK18*mobsacB*, yielding pK18-EryΔR1, pK18-EryΔR2 and pK18-EryΔR1/2. These plasmids were independently mobilized by conjugation to *S. meliloti* Rm1021 to induce double cross-over events as described [9]. Km sensitive and Er resistant colonies were finally checked for the targeted deletion by colony PCR with the primer pairs 3–15C1-i/15C2sec-i, 15C1sec-i/5–15C2-i, and 15C1sec-i/15C2sec-i as well as by Southern and Northern analyses. All the pGEM-T constructs generated throughout the procedure were checked by full sequencing of the cloned inserts.

Plasmids pSRK-R1 and pSRK-R2 constitutively expressing AbcR1 and AbcR2 sRNAs, respectively, were generated by engineering the mid-copy (∼30–40 copies/cell) pBBR1MCS-2 derivative pSRKKm [67] as follows. First, the *Bam*HI site was removed from the polylinker of pSRKKm by *Bam*HI restriction followed by filling the 5′ overhangs with the Klenow enzyme and religation to yield plasmid pSRK*. This plasmid was amplified with the divergent primers FwSRK and RvSRK that remove the binding site of the LacI repressor within the P_lac_ promoter and incorporate a new *Bam*HI site immediately upstream of the *Sac*I site in the polylinker. The PCR product was digested with *Bam*HI and religated to generate pSRK. The full-length *abcR1* and *abcR2* genes (i.e. from the transcription start site to the last residue of the Rho-independent terminator) were amplified by PCR using Rm1021 genomic DNA as template and the primer pairs Smr15C2F/Smr15C2R and Smr15C1F/Smr15C1R that incorporate *Bam*HI and *Sac*I sites to the 5′- and 3′-ends of the fragments, respectively. These PCR products were ligated to pGEM-T Easy to generate, pGEM-R1 and pGEM-R2. The AbcR1 and AbcR2 *loci* were retrieved as *Bam*HI/*Sac*I fragments from pGEM-R1 and pGEM-R2 and inserted downstream the modified P_lac_ promoter in pSRK, yielding plasmids pSRK-R1 and pSRK-R2. All the pSRKKm-derived constructs were checked by sequencing with primer secSRK. pSRK (control plasmid), pSRK-R1 and pSRK-R2 were mobilized to Rm1021 or mutant derivatives as required by conjugation. Constitutive transcription of AbcR1 and AbcR2 from pSRK-R1 and pSRK-R2, respectively, was verified by Northern analysis (Fig. S2).

All PCR amplifications were performed with the proofreading DNA polymerase Phussion® (New England Biolabs, NEB).

### 2D-PAGE Analysis of Periplasmic Proteins

Periplasmic protein fractions were prepared from the *S. meliloti* Rm1021 derivative strains ΔR1(pSRK), ΔR1(pSRK-R1), ΔR2(pSRK) and ΔR2(pSRK-R2) grown in 200 ml of TY broth to exponential phase (OD_600_ 0.6) according to the protocol described in [68]. Proteins were precipitated using the TCA-acetone method [69] and solubilized in free-dithiothreitol (DTT) rehydration solution (8 M urea, 2 M thiourea, 4% CHAPS and traces of bromophenol blue). Protein concentration in the samples was determined by the Bradford assay [70]. For 2D electrophoresis, proteins (1 mg) were solubilized in 250 µl of rehydration solution containing 8 M urea, 2 M thiourea, 4% CHAPS, 1% DTT, 3 µl Deastreak (Amersham), 1.5 µl of IPG buffer and traces of bromophenol blue. The mixture was actively rehydrated on Immobiline DryStrip (13 cm–pH 4 to 7) (Amersham Biosciences) overnight at 50 V and subjected to isoelectric focusing using the following program settings: 30 min at 250 V, ramping 1 h to 8,000 V, and a final phase of 8,000 V until reaching 20,000 W/h. The strips were equilibrated for 15 min by shaking in a solution of 50 mM Tris-HCl pH 8.8 containing 6 M urea, 30% glycerol, 2% SDS and 2% DTT, subjected to a second equilibration for 15 min with the same solution containing 2.5% iodoacetamide and 0.01% of bromophenol blue instead of DTT and then loaded onto 12% polyacrylamide gels. Second-dimension electrophoreses were performed at 35 mA per gel, with a previous 30 min step at 20 mA per gel. Gels were stained with Bio-safe™ Coomassie brilliant blue G-250 (BioRad). Spots corresponding to differentially accumulated proteins were excised from gels, digested with trypsin and subjected to MALDI-TOF MS [proteomics core facilities at Instituto de Parasitología y Biomedicina López Neyra (CSIC) and Universidad de Córdoba]. Protein identification was done with the PRIAM application (http://www.priam.prabi.fr ) and MASCOT program [71].

### Double-plasmid Reporter Assay

To assess *livK* mRNA regulation by AbcR1/2 *in vivo* we used a reporter assay based on that developed by Urban and Vogel for enterobacteria [40]. Our system is based on the co-expression in the same cell of two compatible plasmids transferred by conjugation to the appropriate recipient *S. meliloti* Rm1021 derivative; a mid-copy pBBR1MCS-2 plasmid expressing the full-length sRNA from a modified P_lac_ promoter (the described pSRK series) and a low-copy reporter plasmid (pR*livK::egfp*) derived from the IncP broad host-range vector pJB3Tc19 [72] carrying a translational fusion of the 5′ region of the *livK* mRNA to the N-terminus of EGFP under the control of the constitutive P_syn_ promoter [41].

The reporter plasmid pR*livK::egfp* was constructed as follows. The EGFP coding sequence was amplified by PCR as a *Bam*HI fragment with primers GFPA1 and GFPA2 and plasmid pK7WGF2.0 as template [73]. This PCR product was inserted into the *Bam*HI site of pBBSyn [66] to generate pBBSyn-EGFP. The EGFP ORF along with the P_syn_ promoter was retrieved as a *Hind*III-*Eco*RI fragment by amplification of pBBSyn-EGFP with the primer pair 5SynH/3′-GFP-E and inserted into pGEM-T Easy yielding pGEMPsyn-EGFP. The insert of this plasmid was recovered by *Hind*III/*Eco*RI restriction and ligated to pJB3Tc19, generating pJB_EGFP, which was used as positive control of basal EGFP expression from this vector. pGEMPsyn-EGFP was amplified with the divergent primers Syn-I and GFP-i2 that remove the short 5′-UTR and the ATG start codon of EGFP adding a single *Bam*HI site and contiguous *Bam*HI/*Nhe*I sites to the 3′- and 5′-ends of the PCR product, respectively. The resulting fragment was digested with *Bam*HI and self-ligated to generate pGEMPsyn_GFP-ΔUTR. A Rho-independent transcriptional terminator (T1) was generated by the annealing of primers RhoIT_S and RhoIT_AS that leave protruding ends complementary to the *Sac*I and *Hind*III recognition sequences and inserted between these sites immediately upstream of the P_syn_ promoter in pGEMPsyn_GFP-ΔUTR to generate pGEMPsyn_GFP-ΔUTR-T. The module T1-P_syn_-EGFP was extracted as a *Sac*I-*Eco*RI fragment from this plasmid and ligated to pJB3Tc19, yielding pR-EGFP. Since the *Nhe*I site incorporated in the construct encodes the second and third codons of EGFP any target 5′ region can be translationally fused to the N-terminus of EGFP if cloned into pR-EGFP as *Bam*HI(*Bgl*II)-*Nhe*I fragments. Specifically for this work, the 5′ region of the *livK* mRNA, from its native transcription start site (TSS) to the 16^th^ codon (89 bp), was amplified with the primer pair liv_F/liv_R from Rm1021 genomic DNA and ligated as *Bam*HI-*Nhe*I fragment to pR-EGFP generating plasmid pR*livK::gfp*, which was used as reporter in our assays. Information about the TSS of the *livK* mRNA is derived from RNA-Seq data [74].

All PCR amplifications were performed with the proofreading DNA polymerase Phussion® (NEB) and checked by sequencing.

*S. meliloti* Rm1021 derivatives carrying the different pSRK-pR*livK::egfp* combinations were first grown on TY agar plates during 3 days. Plates were then visualized with a PharosXS scanner (Bio-Rad) at a wavelength of 530 nm and photographed. In addition, total protein extracts from the reporter strains grown to OD_600_ 0.6 in TY broth were subjected to Western blot analysis as described [9], [40]. Membranes were probed with α-GFP polyclonal antibodies (Invitrogen) at a 1∶5,000 dilution to detect the LivK::EGFP fusion protein. α-Mouse-HRP (Sigma) at a 1∶100,000 dilution was used as secondary antibody in these experiments. As loading control GroEL was probed with α-GroEL antisera (Enzo life Sciences) (1∶10,000) and α-rabbit-HRP (Sigma) as secondary antibody (1∶150,000).

### Bioinformatics Tools

Secondary structures of the sRNAs were predicted and represented with the programs RNAfold (http://rna.tbi.univie.ac.at/cgi-bin/RNAfold.cgi) [75] and VARNA [76], respectively. Antisense interactions between AbcR1/2 and the *livK* mRNA were searched for with IntaRNA (http://rna.informatik.uni-freiburg.de:8080/v1/IntaRNA.jsp) [77].

## Supporting Information

Figure S1 **The αr15 co-variance model.** Alignment in Stockholm format of the αr15 sRNAs showing the consensus secondary structure. Each of the stems represented by the structure line # = GC SS_consensus are in a different colour, corresponding the red one to the Rho-independent terminator. Names of *S. meliloti* AbcR1 and AbcR2 sRNAs (formerly Smr15C2 and Smr15C1, respectively) are in blue colour, indicated with a double arrowhead. The *A. tumefaciens* homologs (AbcR1 and AbcR2) are in bold. The conserved anti-Shine Dalgarno (aSD) motif and A/U rich single-stranded sequence stretch are indicated. The variable regions targeted by the 25-mer oligonucleotides used as specific probes to detect AbcR1 and AbcR2 on Northern blots are underlined in red. Predicted αr15 genes (AbcR1/AbcR2) in the main chromosome (denoted by “C” or “CI” in the sRNA names), second chromosome (denoted by “CII”) and plasmids (denoted by “p”) of α-proteobacterial genomes are grouped as indicated to the left. Host genomes are identified as follows: Sm = *S. meliloti* 1021, Smed = *S. medicae* WSM419, Sf = *S. fredii* NGR234, At = *A. tumefaciens* C58, AH13 = *A. sp.* H13-3, ReCIAT = *R. etli* CIAT652, Ar = *A. radiobacter* K84, Rlt2304 = *R. leguminosarum* bv. trifolii WSM2304, Avr15C2 = *A. vitis* S4, Rlv = *R. leguminosarum* bv. viciae 3841, Rlt1325 = *R. leguminosarum* bv. trifolii WSM1325, ReCFN = *R. etli* CFN 42, Ml = *Mesorhizobium loti* MAFF303099, Bc = *B. canis* ATCC 23365, Bs23445 = *B. suis* ATCC 23445, BaS19 = *B. abortus* S19, Bs1330 = *B. suis* 1330, Ba19941 = *B. abortus* bv. 1 str. 9–941, Bma = *B. melitensis* bv. abortus 2308, Bo = *B. ovis* ATCC 25840, Bmi = *B. microti* CCM 4915, Oa = *O. anthropi* ATCC 49188.
(TIF)

Figure S2 **Constitutive AbcR1 and AbcR2 (over)expression.** A) Diagram of the genetic constructs tested to express AbcR1 and AbcR2 from the modified P_lac*_ promoter in pSRK [67]. Relevant restriction sites for cloning of the full-length AbcR1/2 *loci* are indicated. B) Northern hybridization analysis of total RNA extracted from *S. meliloti* Rm1021 wild-type strain (wt) and the transconjugants harboring pSRK-R1 (left panel) and pSRK-R2 (right panel) grown to exponential (LOG) and stationary phases (ST) in TY broth. The last two lanes in each panel correspond to RNA samples from the Rm1021 ΔR1 and ΔR2 deletion mutants transformed with pSRK, pSRK-R1 (R1+) or pSRK-R2 (R2+) as indicated on top (i.e. series of strains which periplasmic proteome was compared; Fig. 5). A co-migrating DNA marker is shown to the left of each panel. The hybridization signal corresponding to the 5S rRNA and the ethidium bromide MOPS-formaldehyde gel with the 23S and 16S RNAs are shown below each panel.
(TIF)

Figure S3 **Nodulation competitiveness of the Rm1021 AbcR1/2 mutants.** Sets of 24 individual alfalfa plants grown hydroponically in test tubes were inoculated with bacterial suspensions consisting of mixtures of two strains; a Rm1021 pGUS3-tagged reporter strain [36] and each of the Rm1021 AbcR1/2 deletion (ΔR1, ΔR2 and ΔR1/2; left pannel) or overexpression (pSRK-R1 and pSRK-R2; right panel) mutants at 1∶1, 10∶1 or 1∶10 ratio, as indicated below the graphs. The final bacterial concentration in the plant grown medium was always 10^6^ cells/ml. Rm1021 and Rm1021 carrying the empty pSRK plasmid were co-inoculated with the reporter strain as the reference of wild-type competitiveness in each series of assays. Roots of inoculated plants were stained for GUS activity 30 days after plants inoculation and nodulation competitiveness of the tested strains in each assay was calculated as the percentage of white nodules counted on roots. Values reported are means of three independent experiments. The standard error is also indicated. Multivariate analysis of variance (MANOVA) did not evidence significative differences among the strains.
(TIF)

Table S1 **Bacterial strains and plasmids.** Name and brief description of the bacterial strains and plasmids used in this study.
(PDF)

Table S2 **Oligonucleotide sequences.** Name and sequences of the oligonucleotides used in this study.
(PDF)
