# Supplementary figures and images for: Independent Activity of the Homologous Small Regulatory RNAs AbcR1 and AbcR2 in the Legume Symbiont Sinorhizobium meliloti
Source: PLoS One. 2013 Jul 15;8(7):e68147. doi: 10.1371/journal.pone.0068147 (PMC3712013; doi:10.1371/journal.pone.0068147)

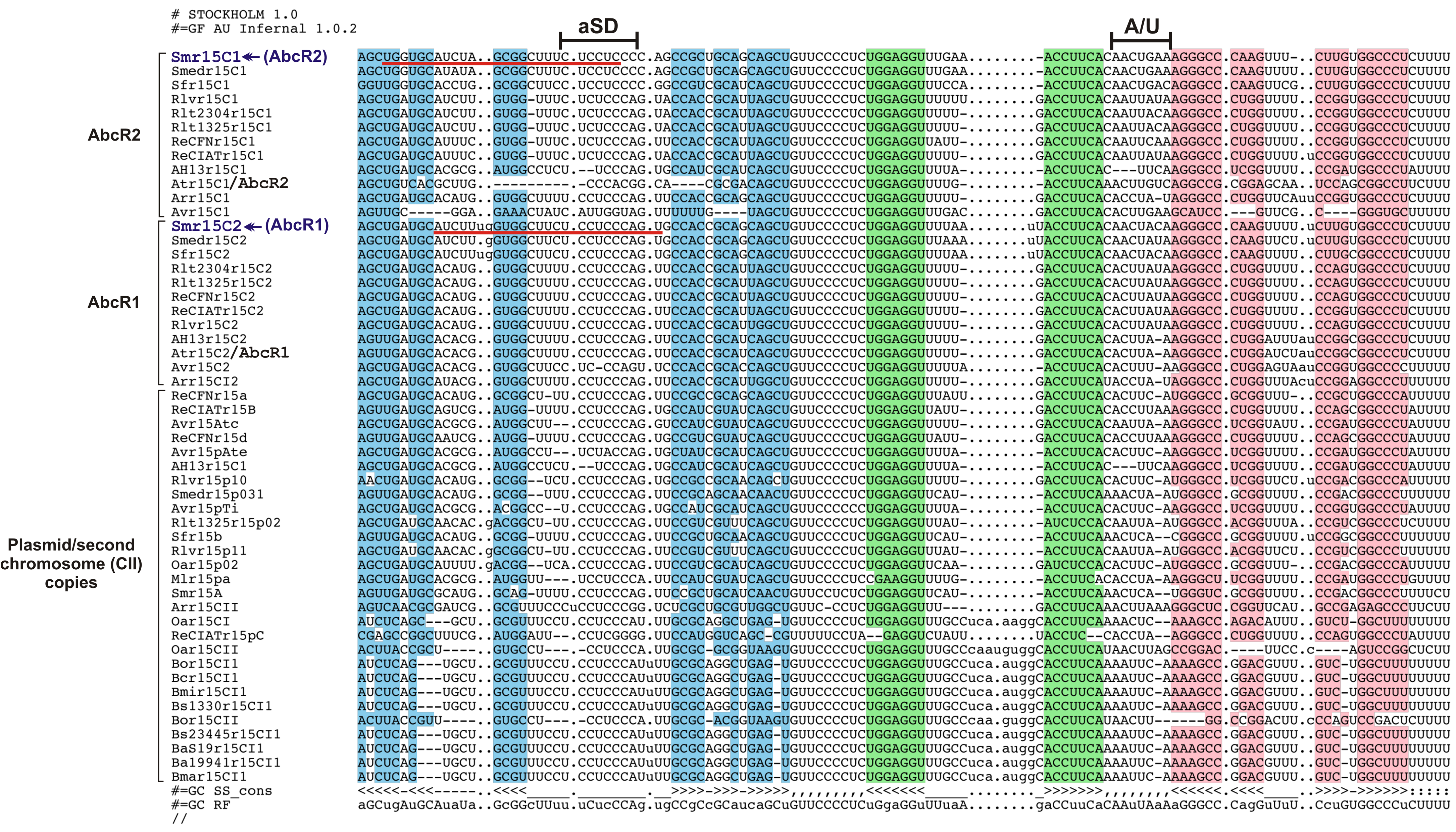

Supplement: Figure S1 — The αr15 co-variance model. Alignment in Stockholm format of the αr15 sRNAs showing the consensus secondary structure. Each of the stems represented by the structure line # = GC SS_consensus are in a different colour, corresponding the red one to the Rho-independent terminator. Names of S. meliloti AbcR1 and AbcR2 sRNAs (formerly Smr15C2 and Smr15C1, respectively) are in blue colour, indicated with a double arrowhead. The A. tumefaciens homologs (AbcR1 and AbcR2) are in bold. The conserved anti-Shine Dalgarno (aSD) motif and A/U rich single-stranded sequence stretch are indicated. The variable regions targeted by the 25-mer oligonucleotides used as specific probes to detect AbcR1 and AbcR2 on Northern blots are underlined in red. Predicted αr15 genes (AbcR1/AbcR2) in the main chromosome (denoted by “C” or “CI” in the sRNA names), second chromosome (denoted by “CII”) and plasmids (denoted by “p”) of α-proteobacterial genomes are grouped as indicated to the left. Host genomes are identified as follows: Sm = S. meliloti 1021, Smed = S. medicae WSM419, Sf = S. fredii NGR234, At = A. tumefaciens C58, AH13 = A. sp. H13-3, ReCIAT = R. etli CIAT652, Ar = A. radiobacter K84, Rlt2304 = R. leguminosarum bv. trifolii WSM2304, Avr15C2 = A. vitis S4, Rlv = R. leguminosarum bv. viciae 3841, Rlt1325 = R. leguminosarum bv. trifolii WSM1325, ReCFN = R. etli CFN 42, Ml = Mesorhizobium loti MAFF303099, Bc = B. canis ATCC 23365, Bs23445 = B. suis ATCC 23445, BaS19 = B. abortus S19, Bs1330 = B. suis 1330, Ba19941 = B. abortus bv. 1 str. 9–941, Bma = B. melitensis bv. abortus 2308, Bo = B. ovis ATCC 25840, Bmi = B. microti CCM 4915, Oa = O. anthropi ATCC 49188. (TIF) [file pone.0068147.s001.tif]

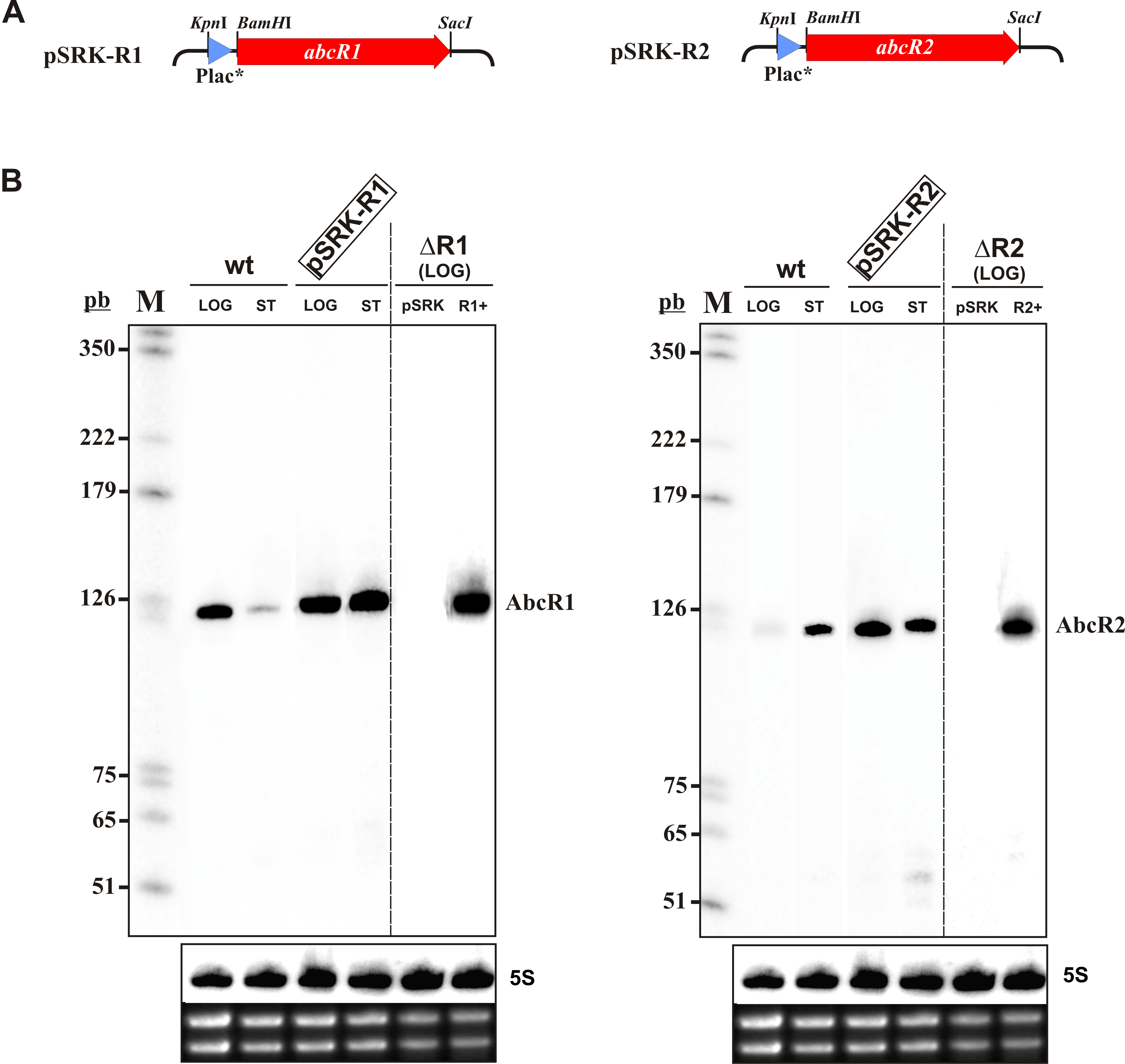

Supplement: Figure S2 — Constitutive AbcR1 and AbcR2 (over)expression. A) Diagram of the genetic constructs tested to express AbcR1 and AbcR2 from the modified Plac* promoter in pSRK [67]. Relevant restriction sites for cloning of the full-length AbcR1/2 loci are indicated. B) Northern hybridization analysis of total RNA extracted from S. meliloti Rm1021 wild-type strain (wt) and the transconjugants harboring pSRK-R1 (left panel) and pSRK-R2 (right panel) grown to exponential (LOG) and stationary phases (ST) in TY broth. The last two lanes in each panel correspond to RNA samples from the Rm1021 ΔR1 and ΔR2 deletion mutants transformed with pSRK, pSRK-R1 (R1+) or pSRK-R2 (R2+) as indicated on top (i.e. series of strains which periplasmic proteome was compared; Fig. 5). A co-migrating DNA marker is shown to the left of each panel. The hybridization signal corresponding to the 5S rRNA and the ethidium bromide MOPS-formaldehyde gel with the 23S and 16S RNAs are shown below each panel. (TIF) [file pone.0068147.s002.tif]

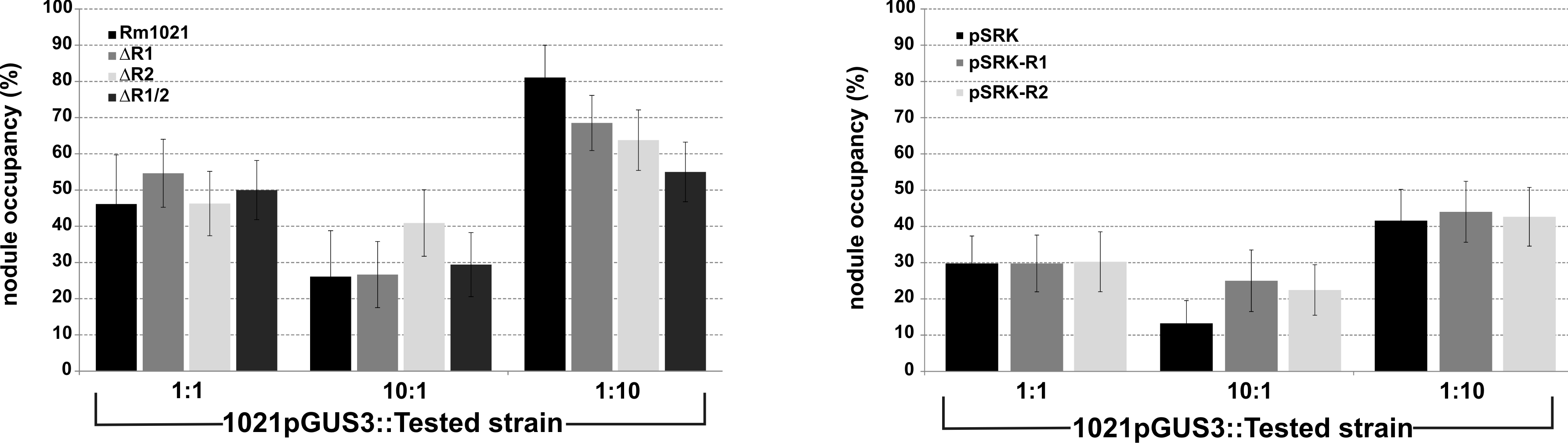

Supplement: Figure S3 — Nodulation competitiveness of the Rm1021 AbcR1/2 mutants. Sets of 24 individual alfalfa plants grown hydroponically in test tubes were inoculated with bacterial suspensions consisting of mixtures of two strains; a Rm1021 pGUS3-tagged reporter strain [36] and each of the Rm1021 AbcR1/2 deletion (ΔR1, ΔR2 and ΔR1/2; left pannel) or overexpression (pSRK-R1 and pSRK-R2; right panel) mutants at 1∶1, 10∶1 or 1∶10 ratio, as indicated below the graphs. The final bacterial concentration in the plant grown medium was always 106 cells/ml. Rm1021 and Rm1021 carrying the empty pSRK plasmid were co-inoculated with the reporter strain as the reference of wild-type competitiveness in each series of assays. Roots of inoculated plants were stained for GUS activity 30 days after plants inoculation and nodulation competitiveness of the tested strains in each assay was calculated as the percentage of white nodules counted on roots. Values reported are means of three independent experiments. The standard error is also indicated. Multivariate analysis of variance (MANOVA) did not evidence significative differences among the strains. (TIF) [file pone.0068147.s003.tif]
