# Supplementary material for: Independent Activity of the Homologous Small Regulatory RNAs AbcR1 and AbcR2 in the Legume Symbiont Sinorhizobium meliloti
Source: PLoS One. 2013 Jul 15;8(7):e68147. doi: 10.1371/journal.pone.0068147 (PMC3712013; doi:10.1371/journal.pone.0068147)
Supplement: Table S1 — Bacterial strains and plasmids. Name and brief description of the bacterial strains and plasmids used in this study. (PDF) [file pone.0068147.s004.pdf]

Table S1. Bacterial strains and plasmids

Strain/Plasmid	Relevant characteristics	Reference
**Bacteria**		
S. meliloti		
Rm1021	Wild-type SU47 derivative, Sm^r	[1]
1021ΔR1	AbcR1 deletion mutant; Er^r, Sm^r	This work
1021ΔR2	AbcR2 deletion mutant; Er^r, Sm^r	This work
1021ΔR1/2	AbcR1/2 double deletion mutant; Er^r, Sm^r	This work
E. coli		
DH5α	F^-, ø80d*lacZ*ΔM15, Δ(*lacZYA-argF*)U169, *deo*R, *recA*1, *endA*1, *hsdR*17(rK^-, mK^+), *phoA*, *supE*44, λ–, *thi*-1, *gyrA*96, *relA*1	Bethesda Research Lab
HB101	*sup*E44, Δ(*mcrC-mrr*), *rec*A13, *ara*-14, *pro*A2, *lac*Y1, *gal*K2, *rps*L20, *xyl*-5, *mtl*- 1, *leu*B6, *thi*-1	Promega Corporation
**Plasmids**		
pRK2013	Helper plasmid, *Col*E1, Km^r	[2]
pGEM®-T Easy	Cloning vector for PCR, Ap^r	Promega Corporation
pSRKKm	pBBR1MCS-2 derivative with a P_{lac} promoter, *lacIq*, *lacZa*+, Km^r	[3]
pK18*mobsacB*	Suicide plasmid in *S. meliloti*, *sacB*, *ori*V, Km^r	[4]
pJB3Tc19	Broad host-range IncP cloning vector, Ap^r, Tc^r	[5]
pBlueScript-KS II	Multi-copy plasmid for *in vitro* transcription, Ap^r	Stratagen
pKS-R1	pBlueScript-KS II with full-length AbcR1 sequence cloned as *Bam*HI-*Sac*I, Ap^r	This work
pKS-R2	pBlueScript-KS II with full-length AbcR2 sequence cloned as *Bam*HI-*Sac*I, Ap^r	This work
pGEMgR1/R2	Genomic region *abcR1/2* inserted into pGEM-T	This work
pGEMΔR1	Genomic region *abcR1/2* with *abcR1* deletion inserted into pGEM-T	This work
pGEMΔR2	Genomic region *abcR1/2* with *abcR2* deletion inserted into pGEM-T	This work
pGEMΔR1/2	Genomic region *abcR1/2* with double *abcR1/2* deletion inserted into pGEM-T	This work
pGEMSSDUTI	pGEM-T with the erythromycin resistance cassette SSDUT1	This work
pGEM-EryΔR1	pGEMΔR1 with SSDUT1 replacing *abcR1*, Er^r	This work
pGEM-EryΔR2	pGEMΔR2 with SSDUT1 replacing *abcR2*, Er^r	This work
pGEM-EryΔR1/2	pGEMΔR1/2 with SSDUT1replacing *abcR1/2*, Er^r	This work
pK18-EryΔR1	pK18*mobsacB* with the pGEM-EryΔR1 insert	This work

Table S1. Bacterial strains and plasmids (Continued)

Strain/Plasmid	Relevant characteristics	Reference
pK18-EryΔR2	pK18*mobsacB* with the pGEM-EryΔR2 insert	This work
pK18-EryΔR1/2	pK18*mobsacB* with the pGEM-EryΔR1/2 insert	This work
pSRK*	pSRKKm lacking the *BamH*I restriction site	This work
pSRK	pSRK* lacking the LacIQ operator	This work
pGEM-R1	pGEM-T Easy with the *abcR1* coding sequence	This work
pGEM-R2	pGEM-T Easy with the *abcR2* coding sequence	This work
pSRK-R1	pSRK_C with the *abcR1* coding sequence	This work
pSRK-R2	pSRK_C with the *abcR2* coding sequence	This work
pK7WGF2.0	Gateway^{TM} derivative plasmid for expression of translational fusion with fluorescent tags	[6]
pGEMPsyn-EGFP	pGEM-T with the EGFP coding sequence under the control of the P_{syn} promoter	This work
pJB_EGFP	pJB3Tc19 with the EGFP coding sequence under the control of the P_{syn} promoter	This work
pGEM_GFP-ΔUTR	pGEM-T with the P_{syn}-EGFP unit	This work
pGEM_GFP-ΔUTR-T	pGEM-T with the P_{syn}-EGFP unit preceded by a T1 transcription terminator	This work
pR_EGFP	Reporter fusion plasmid for cloning of sRNA targets	This work
pR*livK::egfp*	pR_EGFP expressing the *livK::egfp* translational fusion	This work

1. Meade HM, Long SR, Ruvkun GB, Brown SE, Ausubel FM (1982) Physical and genetic characterization of symbiotic and auxotrophic mutants of *Rhizobium meliloti* induced by transposon Tn*5* mutagenesis. J Bacteriol 149: 114-122.
2. Figurski DH, Helinski DR (1979) Replication of an origin-containing derivative of plasmid RK2 dependent on a plasmid function provided in trans. Proc Natl Acad Sci USA 76: 1648-1652.
3. Khan SR, Gaines J, Roop RM, Farrand SK (2008) Broad-host-range expression vectors with tightly regulated promoters and their use to examine the influence of TraR and TraM expression on Ti plasmid quorum sensing. Appl Environ Microbiol 74: 5053-5062.
4. Schafer A, Tauch A, Jager W, Kalinowski J, Thierbach G, et al. (1994) Small mobilizable multi-purpose cloning vectors derived from the *Escherichia coli* plasmids pK18 and pK19: selection of defined deletions in the chromosome of *Corynebacterium glutamicum*. Gene 145: 69-73.
5. Blatny JM, Brautaset T, Winther-Larsen HC, Haugan K, Valla S (1997) Construction and use of a versatile set of broad-host-range cloning and expression vectors based on the RK2 replicon. Appl Environ Microbiol 63: 370-379.
6. Karimi M, De Meyer B, Hilson P (2005) Modular cloning in plant cells. Trends Plant Sci 10: 103-105.
