# Supplementary material for: Independent Activity of the Homologous Small Regulatory RNAs AbcR1 and AbcR2 in the Legume Symbiont Sinorhizobium meliloti
Source: PLoS One. 2013 Jul 15;8(7):e68147. doi: 10.1371/journal.pone.0068147 (PMC3712013; doi:10.1371/journal.pone.0068147)
Supplement: Table S2 — Oligonucleotide sequences. Name and sequences of the oligonucleotides used in this study. (PDF) [file pone.0068147.s005.pdf]

Table S2. Oligonucleotide sequences

Name	Nucleotide sequence
5-15C1cir	5’-CCTCTGGAGGTTTGAAACCT-3’
3-15C1cir	5’-AACAGCTGCTGCAGCGGCTG-3’
5-15C2cir	5’-GTGCCACCGCAGCAGCTGTT-3’
3-15C2cir	5’-TGGGAGGAGAAGCCACCAAA-3’
AbcR2 probe	5’-GAGGAGAAAGCCGCTAGATGCACCA-3’
AbcR1 probe	5’-ACTGGGAGGAGAACGGAGCAAAGAT-3’
5-15C1	5’-GCATGCGGATTGACGAGGTAGGTCTT-3’
3-15C2	5’-TCTAGAGGCTTGATCCTGACCGAACA-3’
5-15C1-i	5’-GAGGTACCTTTGCCCGGCGTTTATGG-3’
3-15C1-i	5’-GAGGTACCATATGGGTAGGGCCGTAG-3’
5-15C2-i	5’-GAGGTACCACAGCCCTCAGTTGCGAA-3’
3-15C2-i	5’-GAGGTACCTTGCAGCGCACAAGGATT-3’
SalSyn	5’-GCGTCGACTATAAAAATAATTCTTGACAT-3’
SynXho	5’-CCGCTCGAGTTAATGGCGCATATTATACC-3’
fwSDU-p	5’-TCGACTTAAGAAGGAGATATACATATGTCCCTCAAGGTCTGATCACC-3’
rvSDU-p	5’-TCGAGGTGATCAGACCTTGAGGGACATATGTATATCTCCTTCTTAAG-3’
5’T1	5’-TGGTCGACGCTAGCAGGCATCAAATAAAACGAAA-3’
3’T1	5’-TACTCGAGAGCGTTCACCGACAAACA-3’
5-Ery-Kpn	5’-GGTACCTATAAAAATAATTCTTGAC-3’
3-Ery-Kpn	5’-GGTACCGAGCGTTCACCGACAAACA-3’
15C1sec-i	5’-TGAGATGAAACCGTCGACTG-3’
15C2sec-i	5’-ACTGGAACTTCTGACGATCC-3’
secSRK	5’-TTCCATTCGCCATTCAGGCT-3’
FwSRK	5’-ACTAAAGGGATCCAAAGCTGGAGC-3’
RvSRK	5’-GCTCACAATTGGATCCAACATACGAG-3’
Smr15C1F	5’-GGATCCAGCTGGTGCATCTAGCGG-3’
Smr15C1R	5’-GAGCTCGCCGGGCAAAATGCCGAC-3’
Smr15C2F	5’-GGATCCAGCTGATGCATCTTTGGT-3’
Smr15C2R	5’-GAGCTCTGGGGCTCCGCCAGGCAA-3’
GFPA1	5’-GGATCCACTAGTATGGTGAGC-3’
GFPA2	5’-GGATCCTCACTTGTACAGCTC-3’
3’-GFP-E	5’-GAATTCTCACTTGTACAGCTCGTCCA-3’
5SynH	5’-AAGCTTATAAAAATAATTCTTGACA-3’
GFP-i2	5’-CCGGATCCGCTAGCAAGGGCGAGGAGCTGTT-3’
Syn-i	5’-CGAGGATCCTATTATACCAAATTTTAGCA-3’
RhoIT_AS	5’-AGCTTACAGGGATAAAAAAAGCAGCACTGTAATCAGTGCTG CTTTGTCGTTTTTTCAGAGCT-3’
RhoIT_S	5’-CTGAAAAAACGACAAAGCAGCACTGATTACAGTGCTGCTTT TTTTATCCCTGTA-3’
livK_F	5’-GGATCCTGGTGCTTCCGTGCAAGCAG-3’
livK_R	5’-GCTAGCCACTGCTGTGATTAGTCCGG-3’
